# Glenoid defect size increases but the bone fragment rarely resorbs in shoulders with recurrent anterior instability

**DOI:** 10.1016/j.jseint.2022.12.010

**Published:** 2022-12-21

**Authors:** Shigeto Nakagawa, Takehito Hirose, Ryohei Uchida, Hiroyuki Nakamura, Tatsuo Mae, Kenji Hayashida, Minoru Yoneda

**Affiliations:** a Department of Orthopaedic Sports Medicine, Yukioka Hospital, Osaka, Osaka, Japan; b Department of Orthopaedic Surgery, Daini Osaka Police Hospital, Osaka, Osaka, Japan; c Department of Orthopaedic Sports Medicine, Kansai Rosai Hospital, Amagasaki, Hyogo, Japan; d Soai Orthopaedic Clinic, Takarazuka, Hyogo, Japan; e Osaka Yukioka Medical University, Ibaraki, Osaka, Japan; f Department of Orthopaedic Surgery, Osaka Central Hospital, Osaka, Osaka, Japan; g Department of Orthopaedic Surgery, Kashiwa Tanaka Hospital, Kashiwa, Chiba, Japan

**Keywords:** Recurrent anterior shoulder instability, Glenoid defect size, Bone fragment size, Bone fragment resorption, Glenoid defect enlargement, Glenoid fracture

## Abstract

**Background:**

With recurrent anterior instability the bone fragment of a bony Bankart lesion is often small compared to the glenoid defect. The purpose of the present study was to clarify the changes to both the bone fragment and glenoid defect over time in a single subject.

**Methods:**

Participants were patients who underwent computed tomography (CT) at least twice after an instability event between 2004 and 2021 and had a fragment-type glenoid at first CT. The glenoid rim width (*A*), glenoid defect width (*B*), and bone fragment width (*C*) were measured in millimeters. If *B* or *C* increased by 1 mm or more from the first to final CT, the change was judged as “enlarged,” and if *B* or *C* decreased by 1 mm or more, it was judged as “reduced”; all other cases were judged as “similar.” Then, glenoid defect size and bone fragment size were calculated as *B/A*×100% and *C/A*×100%, respectively, and the changes from the first to final CT were compared.

**Results:**

From the first to final CT, the glenoid defect was enlarged in 30 shoulders, similar in 13 shoulders, and reduced in 4 shoulders, and the bone fragment was enlarged in 18 shoulders, similar in 24 shoulders, and reduced in 5 shoulders. The mean glenoid defect size significantly increased from 10.9% to 15.3% (*P* < .001), and the mean bone fragment size increased from 6.4% to 7.8%, respectively (*P* = .005). At the final CT, in 6 shoulders a new glenoid fracture was observed at a different site from the original fracture. When they were excluded from the analyses, the mean glenoid defect size still significantly increased (from 11.2% to 15.2%; *P* < .001), but the mean bone fragment size did not (6.5% vs. 7.3%, respectively; *P* = .088).

**Conclusions:**

In shoulders with recurrent anterior instability, glenoid defect size appears to increase significantly over time, whereas the bone fragment size remains unchanged or increases only slightly. Bone fragment resorption is quite rare, and a bone fragment appears to be small because of an enlarged glenoid defect.

In patients with recurrent anterior shoulder instability, Sugaya et al performed detailed 3-dimensional computed tomography (3D-CT) examinations of glenoid bone morphology and found glenoid defects in 90% of the shoulders; 50% of the shoulders had a bony Bankart lesion (fragment type), and 40% had no bone fragment (erosion type).^17^ Burkhart and De Beer considered that patients with a significant bony defect, such as an inverted pear glenoid, were not candidates for arthroscopic Bankart repair (ABR).^1^ A preoperative glenoid defect of 20% to 25% was suggested to be the critical size at which ABR should generally be avoided.^6^^,^^8^^,^^18^ On the other hand, Shaha et al investigated clinical outcomes in active duty military personnel and reported that a preoperative glenoid defect of 13.5% was an appropriate threshold for ‘‘subcritical’’ bone loss, which led to a clinically significant worsening in Western Ontario Shoulder Instability Index scores consistent with an unacceptable outcome.^16^ Recently, a glenoid defect of 13.5% has become accepted as the most common cutoff value. However, Cavalier et al reported that patients with subcritical glenoid bone loss (>10%) and younger age (≤23 years) are at risk of failure and reoperation after ABR with Hill-Sachs remplissage.^2^ Nakagawa et al reported that in male competitive rugby and American football players, the postoperative recurrence rate was low when the glenoid defect size was 5% or less.^12^ Therefore, the amount of subcritical glenoid bone loss as being safe in collision/contact athletes and military personnel might have been lower than 13.5%.

On the other hand, sometimes patients develop recurrent anterior shoulder instability, yet have a bone fragment that appears small compared with the size of the glenoid defect (Fig. 1). In a study on small bone fragments in patients with bony Bankart lesions, Nakagawa et al investigated the correlation between the time after primary trauma and the bone fragment size and found that, within 1 year of the primary event, the bone fragment displayed a marked decrease in size relative to the glenoid defect.^13^ They concluded that most bone fragments undergo extensive resorption in the first year after the primary trauma. In contrast, several authors have reported that the glenoid defect may be enlarged by damage due to recurrent dislocation and subluxation,^5^^,^^9^^,^^15^ so the bone fragment may appear smaller because the glenoid defect itself has become larger. Although there is no doubt that the mismatch in size between the glenoid defect and bone fragment increases with more dislocations and subluxations and a longer period after the primary trauma, the progression of the mismatch has not been clearly elucidated.

**Figure 1:**
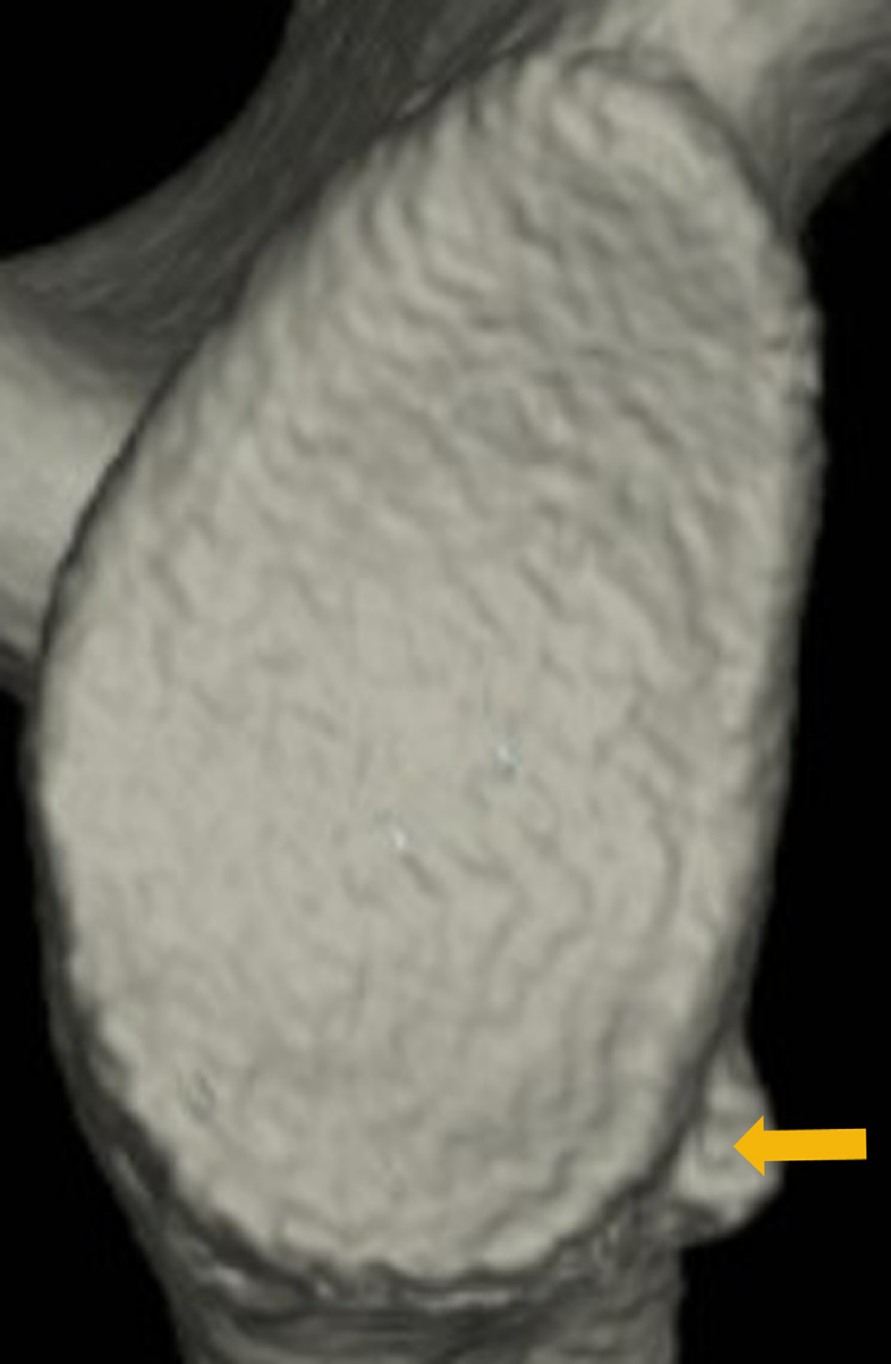
The mismatch between glenoid defect size and bone fragment size in a right shoulder with recurrent anterior instability. A bone fragment appears small compared with the size of the glenoid defect (: bone fragment).

Nakagawa et al reported that if the residual bone fragment was small, the postoperative bone union rate after arthroscopic bony Bankart repair (ABBR) was lower, bone union was delayed, and the postoperative recurrence rate was significantly higher.^14^ Therefore, they suggested that it is important to repair a bony Bankart lesion as soon as possible before the bone fragment size decreases. However, whether a bone fragment size decreases with time is still unclear because Nakagawa et al investigated bone fragment size only once and not over time.^13^

In the present study, we included patients who underwent a CT examination at least twice after an instability event and investigated the changes in glenoid defect size and bone fragment size from the first to final CT. The purpose of the study was to clarify the changes to both the bone fragment and glenoid defect over time in a single subject. We hypothesized that both bone fragment resorption and glenoid defect enlargement develop after several instability events.

## Materials and methods

This was a retrospective case series of prospectively collected clinical data. The study was approved by the local institutional review board, and participants gave written informed consent to participate. Participants were patients who underwent conservative treatment after traumatic anterior shoulder instability (not restricted to patients with primary instability) and experienced further recurrence of instability prior to stabilization surgery in the period from July 2004 to December 2021 and had a fragment-type glenoid at first CT.^17^ They also underwent serial CT examinations at least twice after an instability event. Patients were excluded if they underwent no or only 1 CT examination despite recurrent instability or if they had undergone previous stabilization surgery. Patients with a normal or erosion-type glenoid at first CT were also excluded.

First CT examination was performed at the first hospital visit and second or third CT examination was performed after further recurrence of instability. Patients whose instability recurred more than 6 months after the first CT examination were evaluated by CT again, but to avoid radiation exposure, CT evaluation was usually not performed in patients whose instability recurred within 6 months, and magnetic resonance imaging evaluation was performed alternatively. CT scanning and reconstruction of images were performed with a whole body scanner (spiral scan, 0.5-mm slice thickness, 0.3-mm reconstruction, and 3D edit mode). CT data were analyzed in Digital Imaging and Communications in Medicine mode with Digital Imaging and Communications in Medicinesoftware to perform multiplanar reconstruction.

**Figure 2:**
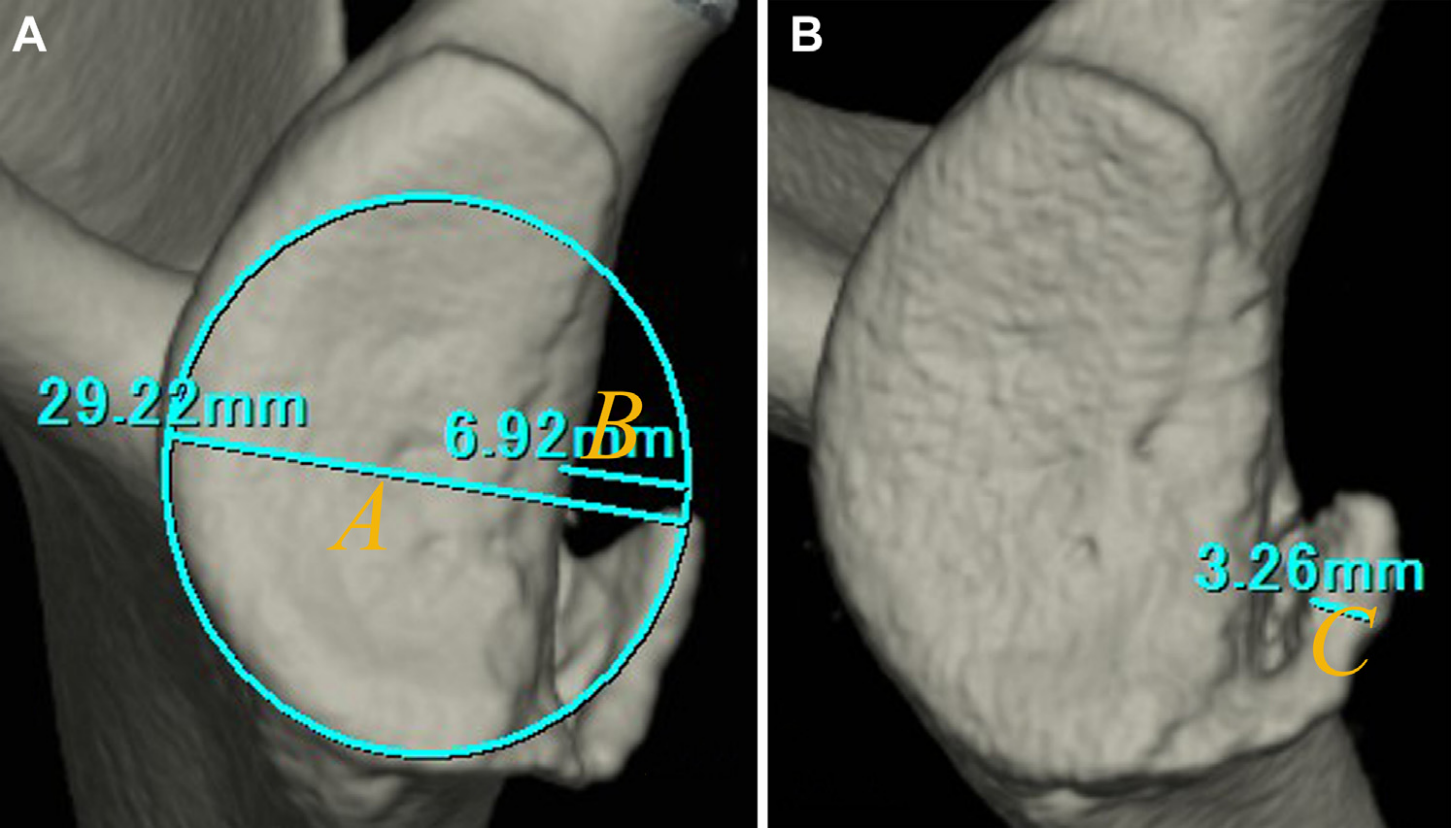
Quantification method for the glenoid defect size and bone fragment size. **(A)**: The glenoid defect size: *B/A*×100%. **(B)**: The bone fragment size: *C/A*×100%. A: the glenoid rim width, B: the width of the glenoid defect, *C*: the width of the bone fragment.

To quantify the glenoid defect, the inferior portion of the glenoid rim was approximated to a true circle on en face 3D-CT scans that were reconstructed by eliminating the head of the humerus. First, the glenoid rim width (*A*) and width of the glenoid defect (*B*) were measured in millimeters, and then, in accordance with the method by Nakagawa et al,^12^^,^^14^ the width of the bone fragment (*C*) was measured in millimeters on the image that gave the clearest view of the articular surface of the fragment. The extent of the glenoid defect was calculated as a percentage of the glenoid rim width (glenoid defect size: *B/A*×100%), and the size of the bone fragment at the widest portion was defined relative to the glenoid rim (bone fragment size: *C/A*×100%) (Fig. 2). We determined the intraobserver reliability by comparing 2 intraobserver measurements performed 1 month apart, and the inter-observer reliability was determined by comparing measurements by 2 independent observers.

For patients who underwent CT twice, glenoid defect size and bone fragment size at the second CT were evaluated. For patients who underwent CT 3 times, the evaluation was performed at the third CT. If the glenoid defect size (*B*) or bone fragment size (*C*) at the final CT had increased by 1 mm or more since the first CT, the change was judged as “enlarged,” and if they had decreased by 1 mm or more, the change was judged as “reduced,” and the other cases were judged as “similar.” The changes in glenoid defect size and bone fragment size from the first to final CT were analyzed.

### Statistical analysis

Mean values were compared by Student’s t test or Mann-Whitney U test, and ratios were compared by Fisher’s exact probability test. When the *P* value was less than .05, a post hoc power analysis was performed. If the study had sufficient statistical power (1-β ≥ 0.8), we determined the difference to be significant.

To investigate the intra- and inter-observer reliabilities of measurements of the glenoid defect and bone fragment size, we determined the intraclass correlation coefficient (1, 1) and interclass correlation coefficient (2, 1); a value greater than or equal to 0.8 was considered to indicate good reliability.

## Results

### Demographics

During the study period, CT examination was performed in 113 patients at least twice after an instability event. At first CT, glenoid rim morphology was normal in 49 shoulders, erosion type in 17 shoulders, and fragment type in 47 shoulders. Among these shoulders, as 66 shoulders with a normal glenoid or an erosion-type glenoid defect were excluded, 47 shoulders with a fragment-type glenoid defect were included in the present study. CT examination was performed twice in 40 of these patients (4 patients were affected bilaterally) and 3 times in 3 of them. The profile of these 47 patients is shown in Table I. Stabilization surgery after final CT was performed in 40 of the 47 patients.

**Table I:** Patients profile.

Total	Patients	*P* value
	47	
Gender		
Male	40	
Female	7	
Affected side		
Right	27	
Left	20	
Type of sports		
Rugby	15	
American football	11	
Martial arts/Judo	6	
Soccer	5	
Basketball	3	
Handball	1	
Badminton	1	
Ski	1	
Snow boding	1	
Fireman	1	
Seizure	1	
No sports	1	
Age at primary instability	19.4 ± 9.2 yr (12-36 yr)	
Age at computed tomography (CT)		<.001
At first CT	21.3 ± 9.1 yr (13-37 yr)	
At final CT	22.8 ± 9.7 yr (14-42 yr)	
Period since primary instability		<.001
At first CT	1.7 ± 3.9 yr (0-23 yr)	
At final CT	3.1 ± 4.6 yr (0-26 yr)	
Total instability events		<.001
At first CT	11.1 ± 23.5 (1-120)	
At final CT	20.8 ± 32.4 (2-150)	
Glenoid defect size		<.001
At first CT	10.9 ± 6.5% (0.8-26.6%)	
At final CT	15.3 ± 6.2% (3.7-28.4%)	
Bone fragment size		.005
At first CT	6.4 ± 3.9% (0.8-17.9%)	
At final CT	7.8 ± 4.6% (1.3-21.1%)	
The ratio of the bone fragment width to the glenoid defect width		<.001
At first CT	65.9 ± 27.5% (12.2-100%)	
At final CT	51.1 ± 25.0% (9.5-100%)	

### Changes in glenoid defect size and bone fragment size from the first to final CT

**Figure 3:**
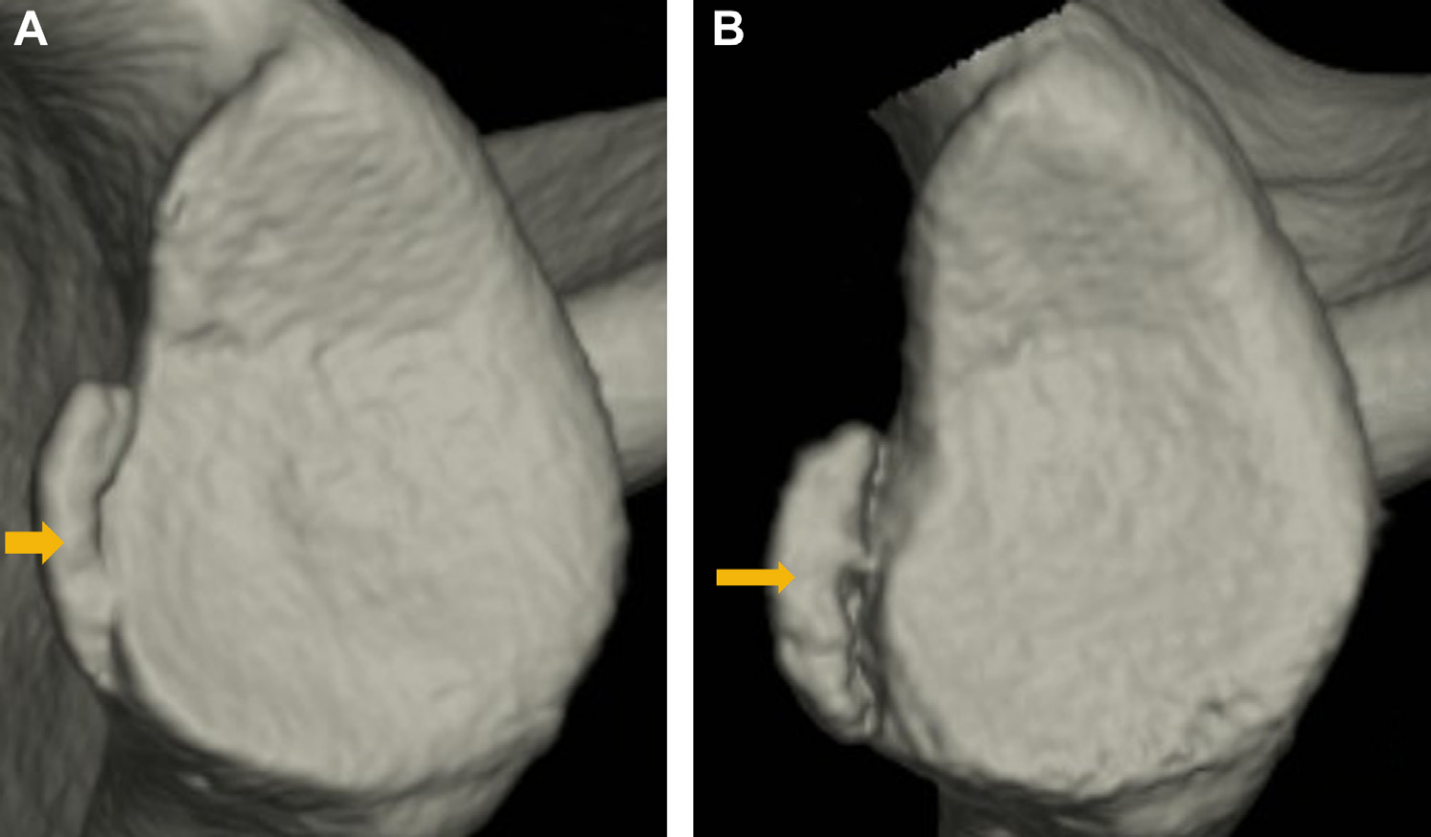
A male soccer player with an enlarged glenoid defect and enlarged bone fragment (left shoulder). **(A)**: First computed tomography (CT) at primary instability (16 years old). **(B)**: Third CT at 2 years after primary instability (18 years old, total 13 instability events). Both a glenoid defect and a bone fragment () increased in size from the first to third CT.

**Figure 4:**
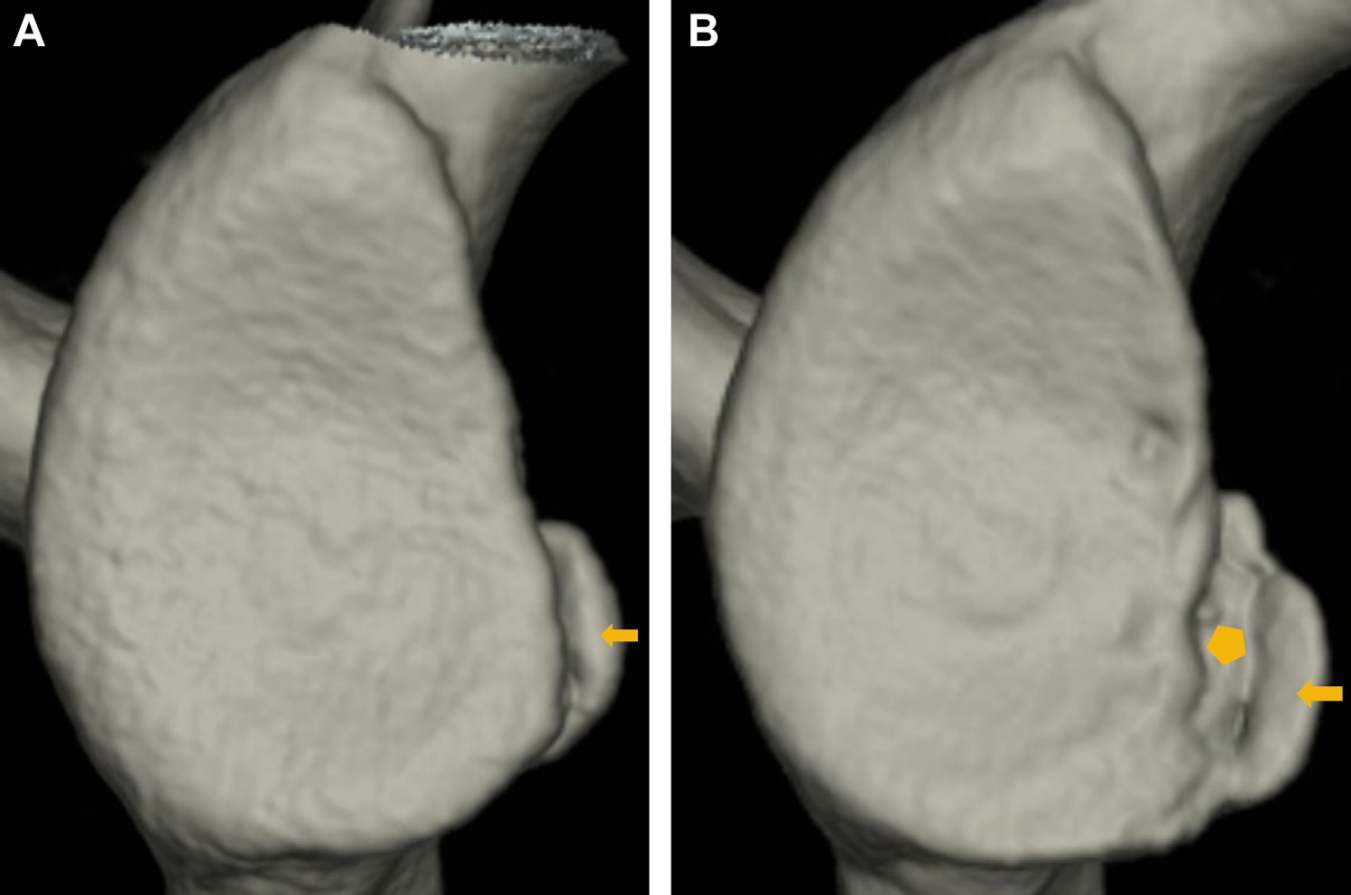
An American football player with a new glenoid fracture at second CT (right shoulder). **(A)**: First CT at 6 months after primary instability (16 years old, once instability event). **(B)**: Second CT at 4 years after primary instability (20 years old, total 4 instability events). At the first CT, he came to our hospital because of instability in left shoulder and did not complain an apprehension in right shoulder. At the second CT, he complained of an apprehensive sensation in right shoulder. A new glenoid fracture () accompanied with a previous bone fragment () was recognized. *CT*, computed tomography.

From the first to final CT, the glenoid defect was enlarged in 30 shoulders, similar in 13 shoulders and reduced in 4 shoulders, and the bone fragment size was enlarged in 18 shoulders, similar in 24 shoulders and reduced in 5 shoulders (Fig. 3). The mean glenoid defect size significantly increased from 10.9% at first CT to 15.3% at final CT (*P* < .001), and the mean bone fragment size increased from 6.4% to 7.8%, respectively (*P* = .005). At the final CT, a new glenoid fracture was observed in 6 shoulders at a different site from the original fracture (Fig. 4), and in all of these shoulders both the glenoid defect and the bone fragment increased in size from the first to final CT (Table II). When these 6 shoulders were excluded from the analyses, the mean glenoid defect size still significantly increased from 11.2% at first CT to 15.2% at final CT (*P* < .001), but the mean bone fragment size was 6.5% and 7.3%, respectively (*P* = .088). The intraobserver reliabilities for the measurement of glenoid defect size and bone fragment size were 0.985 and 0.953, respectively, and the interobserver reliabilities were 0.914 and 0.845, respectively

**Table II:** The changes in glenoid defect size and bone fragment size from the first to final CT.

Glenoid defect	Bone fragment	No. of patients
Enlarged	Enlarged	14 (including 6 patients with a new glenoid fracture)
	Similar	16
	Reduced	0
Similar	Enlarged	4
	Similar	6
	Reduced	3
Reduced	Enlarged	0
	Similar	2
	Reduced	2

*CT*, computed tomography.

We investigated the changes in bone fragment size in each type of glenoid defect change (Table II). In shoulders with an enlarged glenoid defect, bone fragments were enlarged or similar, and in shoulders with a reduced glenoid defect, they were reduced or similar. In the 30 shoulders with an enlarged glenoid defect, the mean glenoid defect size significantly increased from 8.6% at first CT to 15.8% at final CT (*P* < .001), and the bone fragment size also significantly increased from 5.4% to 8.0%, respectively (*P* < .001). When the 6 shoulders with a new glenoid defect were excluded, the mean glenoid defect size still significantly increased from 8.7% at first CT to 15.9% at final CT (*P* < .001), and the mean bone fragment size still increased from 5.3% to 7.1%, respectively (*P* = .002). On the other hand, in the 17 shoulders with a similar or reduced glenoid defect, the mean glenoid defect size was 14.8% at first CT and 14.3% at final CT (*P* = .363), and the mean bone fragment size was 8.1% and 7.7%, respectively (*P* = .578) (Table III).

**Table III:** The changes in bone fragment size in each type of glenoid defect change.

	Glenoid defect size	Bone fragment size
Enlarged glenoid defect (n = 30)		
At first computed tomography (CT)	8.6 ± 4.9%	5.4 ± 3.5%
At final CT	15.8 ± 5.9%	8.0 ± 5.0%
*P* value	<.001	<.001
Similar glenoid defect (n = 13)		
At first CT	13.0 ± 6.5%	7.0 ± 3.4%
At final CT	13.4 ± 6.7%	7.0 ± 4.5%
*P* value	.184	.999
Reduced glenoid defect (n = 4)		
At first CT	20.8 ± 6.4%	11.9 ± 4.3%
At final CT	17.5 ± 6.7%	9.8 ± 1.1%
*P* value	.047	.292
Enlarged glenoid defect excluding a postoperative glenoid fracture (n = 24)		
At first CT	8.7 ± 5.2%	5.3 ± 3.6%
At final CT	15.9 ± 6.4%	7.1 ± 5.0%
*P* value	<.001	.002
Reduced or similar glenoid defect (n = 17)		
At first CT	14.8 ± 7.1%	8.1 ± 4.1%
At final CT	14.3 ± 6.7%	7.7 ± 4.1%
*P* value	.363	.578

*CT*, computed tomography.

## Discussion

The most important finding of the present study was that in shoulders with recurrent anterior instability, the glenoid defect size significantly enlarged from the first to final CT, whereas the bone fragment size remained unchanged or increased slightly. Bone fragment resorption was quite rare, and a bone fragment appeared to be small because of an enlarged glenoid defect, contrary to our hypothesis.

Nakagawa et al reported that bone fragment resorption progresses soon after the primary instability^13^. They measured glenoid defect width (*B*) and bone fragment width (*C*) and calculated the extent of bone fragment resorption as a percentage with the following equation: (1–*C/B*) × 100%. However, they measured bone fragment to glenoid defect ratio at one time point per patient without any follow-up CTs. This data was then compared among subjects to determine if time from instability affected ratio. Therefore, they could not rule out the possibility that the bone fragment may have appeared to be small because the glenoid defect itself had increased in size through damage due to recurrent dislocation and subluxation. In the present study, we evaluated glenoid defect size and bone fragment size at least twice, so we could evaluate the changes in detail. We found that the mean *C:B* ratio was 65.9% at the first CT (mean period after primary instability: 1.7 years) and decreased to 51.1% at the final CT (mean period: 3.1 years) (Table II). According to the criteria of Nakagawa et al,^13^ the extent of bone fragment resorption in the present study was 34.1% at the first CT and 48.9% at the final CT, so our results support their hypothesis that bone fragment resorption progresses over time. However, they defined a decrease in the ratio of *C* to *B* as bone fragment resorption, which is not an appropriate definition because the present study clearly showed that bone fragment resorption was quite rare.

On the other hand, the significant increase in the size of glenoid defects from the first to final CT was compatible with our hypothesis and consistent with several previous reports.^5^^,^^9^^,^^15^ Nakagawa et al reported that the glenoid defect was significantly enlarged by damage due to recurrent dislocations and subluxations.^15^ Nakagawa et al recently reported that glenoid defect size was affected not by the period since primary instability but by the number of instability events.^10^ In the present study, the mean glenoid defect size significantly increased from 10.9% at first CT (mean instability events: 11.1) to 15.3% at final CT (mean events: 20.8). Etoh et al reported that an erosive-type glenoid defect was not a bony erosion caused by repetitive dislocation but rather a compression fracture caused by a single dislocation, in which the humeral head was compressed against the anterior rim of the glenoid.^3^ Nakagawa et al also reported that a small bone fragment may be present in a shoulder with a small glenoid defect at primary instability but that such a fragment may also be compressed on an enlarged glenoid defect after multiple dislocations.^11^ We think that in most patients with recurrent anterior instability, such a mechanism might induce the progression of the size mismatch. However, even if bone fragment resorption does not occur, early stabilization surgery should be recommended before the glenoid defect increases in size because a large glenoid defect and/or a small bone fragment are among the most important risk factors after arthroscopic Bankart repair.

**Figure 5:**
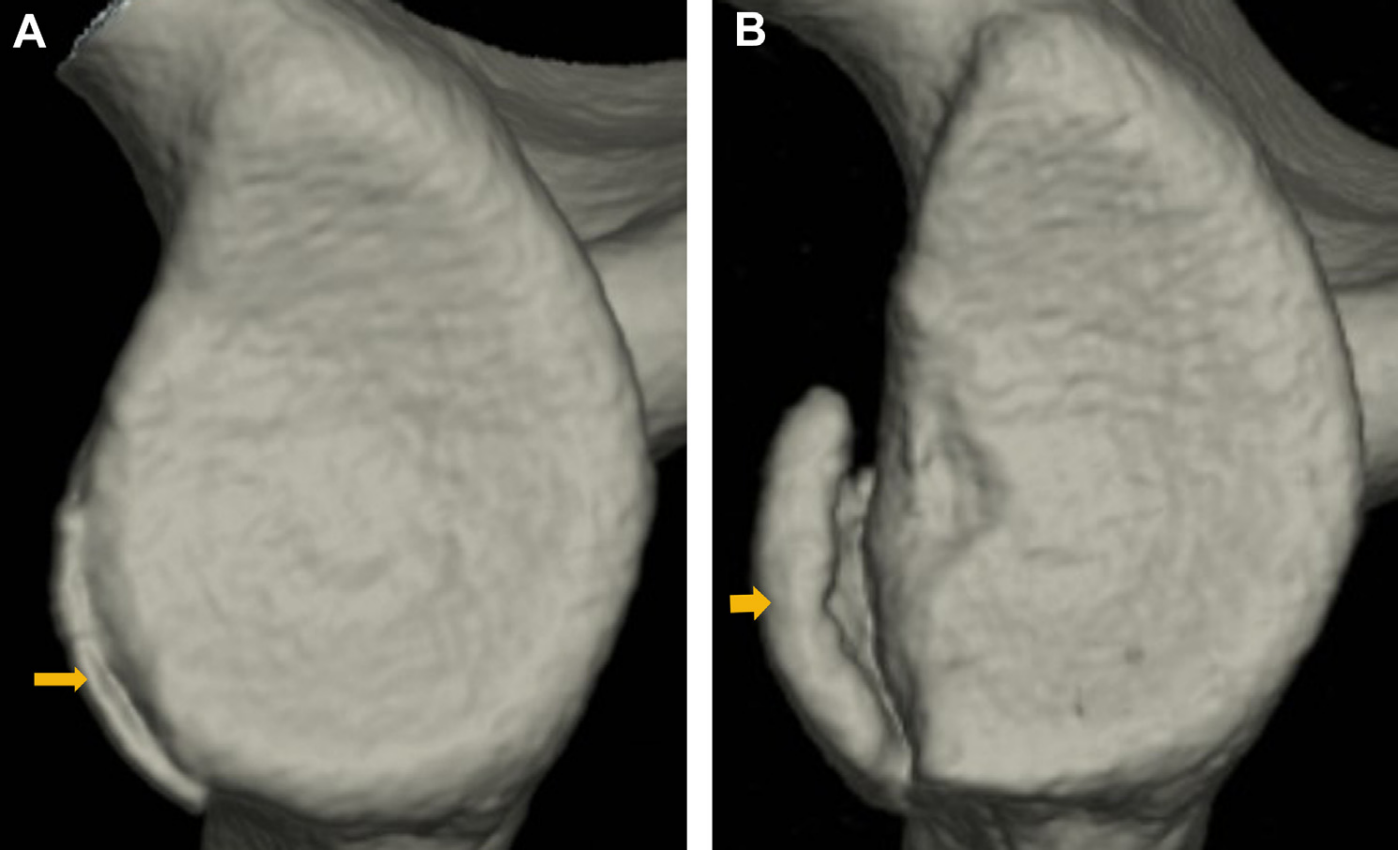
A male rugby player with an enlarged glenoid defect and enlarged bone fragment (left shoulder). **(A)**: First CT at 1 month after primary instability (17 yr old, once instability event). **(B)**: Second CT at 11 months after primary instability (18 yr old, total 10 instability events). At the first CT, a thin bone fragment was recognized. At the second CT, both a glenoid defect and bone fragment increased in size, while the possibility of a new glenoid fracture occurred after complete union of a primary bone fragment could not be denied. (: bone fragment). *CT*, computed tomography.

Contrary to our hypothesis, bone fragment enlargement and not bone fragment resorption was sometimes seen at the final CT. Fujii et al reported that the blood supply persists from the glenoid labrum to the bone fragment of a bony Bankart lesion,^4^ so the healing response after a glenoid fracture might lead to enlargement of the bone fragment. Furthermore, although bone fragments were enlarged or similar in shoulders with an enlarged glenoid defect, they were reduced or similar in shoulders with a reduced glenoid defect (Table III). The reason for this phenomenon is unknown, but Kitayama et al and Nakagawa et al reported that the morphologic characteristics of the glenoid rim become closer to normal after ABBR because the united bone fragment is frequently enlarged by postoperative remodeling.^7^^,^^14^ Although the present study was completely different from these studies, which examined changes after ABBR, in patients without ABBR a similar bone remodeling process that normalizes glenoid rim morphology might occur according to Wolff’s law; in this case, an enlarged glenoid defect might be advantageous for remodeling of a bone fragment. On the other hand, when we excluded the 6 shoulders with a new glenoid fracture at the final CT from the analyses, we found no significant bone fragment enlargement. Because both the glenoid defect and the bone fragment enlarged from the first to the final CT in all 6 of these shoulders, these shoulders might have influenced the finding of bone fragment enlargement at the final CT. Furthermore, besides these 6 patients with a new glenoid fracture, the possibility of a new glenoid fracture could not be ruled out in several patients, so in these patients, a bone fragment accompanied by a minor glenoid fracture might have appeared to be enlarged (Fig. 5). Thus, we might have to conclude that bone fragment size was maintained even after several instability events.

### Limitations

The present study has some limitations, including the retrospective design. The small number of patients did not allow statistical analysis to be performed because of insufficient power. It might be possible that as the bone fragment had a different angulation between the first and final CT, the measurement of bone fragment size was affected by the angulation. However, the width of the bone fragment was measured on the image that gave the clearest view of the articular surface of the fragment. Furthermore, as the change was judged as “enlarged” or “decreased” when bone fragment size at the final CT had increased or decreased by 1 mm or more, minimal error could be ignored.

## Conclusions

In shoulders with recurrent anterior instability, glenoid defect size appears to increase significantly over time, whereas the bone fragment size remains unchanged or increases only slightly. Bone fragment resorption is quite rare, and a bone fragment appears to be small because of an enlarged glenoid defect.

## Disclaimers

Funding: No funding was disclosed by the authors.

Conflicts of interest: The authors, their immediate families, and any research foundation with which they are affiliated have not received any financial payments or other benefits from any commercial entity related to the subject of this article.
